# Tracing the history of cell types

**DOI:** 10.7554/eLife.90447

**Published:** 2023-08-02

**Authors:** Antonia Grausgruber, Roger Revilla-i-Domingo

**Affiliations:** 1 Department of Neuroscience and Developmental Biology, University of Vienna Vienna Austria; 2 Research Platform Single Cell Regulation of Stem Cells, University of Vienna Vienna Austria; 3 Max Perutz Labs, University of Vienna Vienna Austria

**Keywords:** patiria miniata, novelty, single nuclei, transcriptomics, cell type, evolution, echinoderm, sea urchin, sea star, None

## Abstract

A study of sea urchin and sea star larvae paves the way for understanding how cell types evolve and give rise to novel morphologies.

**Related research article** Meyer A, Ku C, Hatleberg WL, Telmer CA, Hinman V. 2023. New hypotheses of cell type diversity and novelty from orthology-driven comparative single cell and nuclei transcriptomics in echinoderms. *eLife* **12**:e80090. doi: 10.7554/elife.80090.

For decades scientists have been fascinated with how evolution gave rise to the wide range of shapes and sizes that exist in the animal kingdom today. At the heart of this morphological variation are the different cell types that make up an organism, some of which will be unique to certain species (Arendt et al., 2016). However, how new cell types emerged over the course of evolution is still hotly debated.

A group of animals known as echinoderms – which includes sea urchins, sea stars and brittle stars among others – offer a fascinating example of morphological novelty. Although their larvae share many morphological features, there are also some clear distinctions, making them a useful system for studying how specific cell types evolved. For instance, the two most well-studied echinoderms, sea stars and sea urchins, develop in a remarkably similar way. However, despite this, sea urchins have two cell types that are not found in sea stars (Figure 1A): skeletogenic mesenchyme cells, which make the mineral skeleton that gives sea urchin larvae their distinct shape, and pigmented cells that reside in the outer epithelium and are recruited to the immune system during an infection (Decker and Lennarz, 1988; Gustafson and Wolpert, 1967; Buckley and Rast, 2017). Now, in eLife, Veronica Hinman and colleagues from Carnegie Mellon University – including Anne Meyer as first author – report new insights in to how these two cell types emerged over the course of evolution (Meyer et al., 2023).

**Figure 1.**
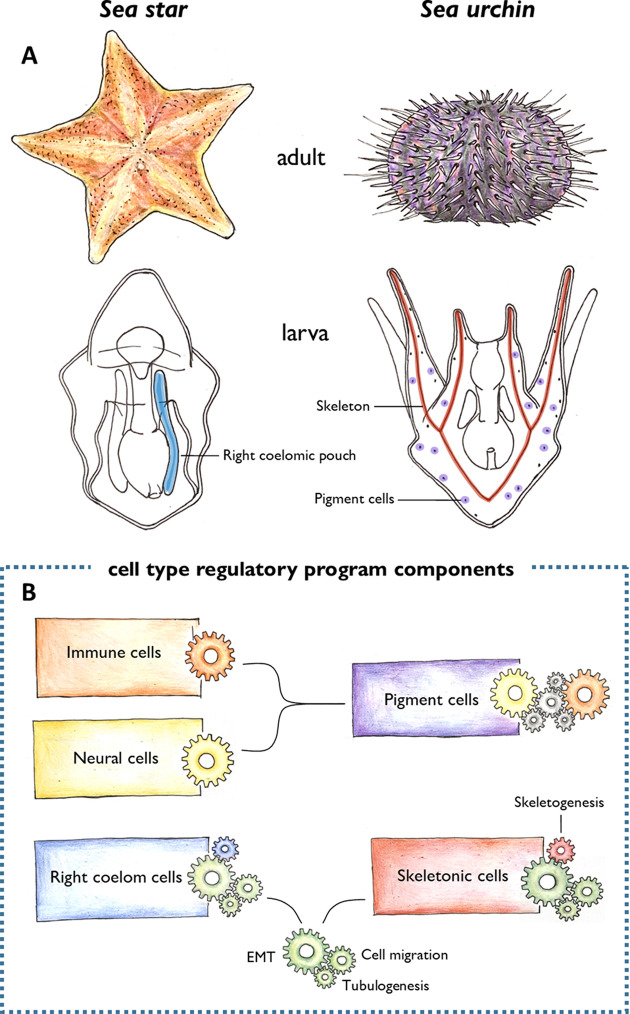
Morphological novelties in the sea urchin larva. (**A**) Schematic representation of the adult (top) and larval (bottom) forms of sea stars and sea urchins. Two larval cell types had been thought to be unique to the sea urchin: the skeletogenic mesenchyme cells that make the skeleton (red), and the pigment cells embedded in the larval epithelium (purple). Meyer et al. found that the skeletogenic mesenchyme cells express similar genes to the right coelomic pouch of the sea star larva (blue), and the pigment cells are similar to immune and neural cells of the sea star larva (not shown). (**B**) These cells were found to share regulatory mechanisms (represented by different coloured clockwork wheels). Pigment cells activate similar regulatory components as immune (orange wheel) and neural (yellow wheel) cells in sea stars. Unknown components (grey wheels) may control the interplay between the immune and neural functions of these cells. The right coelomic pouch of the sea star turns on regulatory components required to produce mesenchymal cells (green wheels) – epithelial to mesenchymal transition (EMT), cell migration, and tubulogenesis – which are also activated in skeletogenic mesenchyme cells in sea urchins. This suggests that skeletogenic mesenchyme cells and cells of the right coelomic pouch evolved from the same ancestor. Later in evolution, the skeletogenic mesenchyme cells acquired a skeletogenic program (red wheel), while the right coelomic pouch cells might have acquired other currently unknown programs (blue wheel).

Image credit: Antonia Grausgruber.

Cell types are typically defined based on their morphology, function, and where and when in the embryo they originated. But Meyer et al. propose that analysing these attributes alone is not sufficient for tracing how a cell type evolved. Instead, the team focused on the inner machinery that regulates all attributes of every cell. They did this by employing a technique called single-nuclei RNA sequencing, which reveals the genes expressed in every cell of an organism. This information can then be used to determine which cells activate similar regulatory mechanisms and therefore might be evolutionary related (Marioni and Arendt, 2017; Tanay and Sebé-Pedrós, 2021).

Meyer et al. used this powerful method to see how cells in the larva of a sea star compare to previously published sequencing data from the larva of a sea urchin (Foster et al., 2020). This revealed a very different picture than expected: both cell types that were thought to be unique to sea urchins appeared to have counterparts in the sea star larva (Figure 1A and B).

The skeletogenic mesenchyme cells of the developing sea urchin were similar to cells of the right coelomic pouch in the sea star which buds off the top of the embryonic structure that gives rise to the gut (Child, 1941). Many of the genes they shared are known to regulate the developmental processes required to generate mesenchymal cells, including epithelial to mesenchymal transition (EMT), cell migration and tubulogenesis. This suggests that skeletogenic mesenchyme cells and cells in the right coelomic pouch evolved from a common ancestor, which also possessed these regulatory mechanisms (Figure 1B). It is likely that these cells then evolutionarily diverged to activate the sets of genes responsible for building the skeleton in sea urchins (Gao and Davidson, 2008).

The pigment cells of the sea urchin were also found to be similar to immune and neural cell types in the sea star. Based on the genes shared between them, Meyer et al. propose that pigment cells have a dual role (immune and neural) in sea urchin larvae, while these functions are exerted by two separate cell types in sea stars (Figure 1B). Although a specific evolutionary history for these cell types is not provided in this study, these results support previous findings suggesting that immune and neural cells are evolutionarily related (Klimovich and Bosch, 2018).

A number of questions still remain about the evolutionary history of skeletogenic mesenchyme and pigment cells. For example, while the pigment cells seem to be unique to sea urchins, a larval skeleton is also present in other echinoderms, such as brittle stars. Extending the approach used by Meyer et al. to more echinoderms will provide a deeper understanding of how the cell types that give rise to the larval skeleton emerged. In addition, unravelling the networks of genes that control the development of skeletogenic mesenchyme and pigment cells in various echinoderms will help reveal the precise events that led to these evolutionary novelties. The findings of Meyer et al. provide a very fertile ground from which to address these questions, and more broadly how the enormous diversity of animal forms arose during evolution.
